# Social Sensing and Individual Brands in Sports: Lessons Learned from English-Language Reactions on Twitter to Pau Gasol’s Retirement Announcement

**DOI:** 10.3390/ijerph20020895

**Published:** 2023-01-04

**Authors:** Jairo León-Quismondo

**Affiliations:** Faculty of Sport Sciences, Universidad Europea de Madrid, 28670 Madrid, Spain; jairo.leon@universidadeuropea.es

**Keywords:** social sensing, sport, Twitter, content analysis, retirement, individual-level brand

## Abstract

Pau Gasol announced his retirement on 5 October 2021. Subsequently, a number of users virtually reacted. Twitter is one of the most popular social media platforms, with more than 368 million active users, generating large-scale social data. This study used data from Twitter for analyzing social sensing related to an individual brand, Pau Gasol’s retirement announcement, from a quantitative and qualitative content analysis perspective. Pau Gasol’s farewell can be considered a unique event to which many people are emotionally attached, providing a great opportunity for understanding sports virtual ecosystems. A total of 2089 tweets in the English language were recovered from Tuesday 5 October 2021 at 3:00 to Thursday 7 October 2021 at 23:59, Greenwich Mean Time +00:00 time zone. During this time, posts were observed to be mainly influential during and right after Pau Gasol’s ceremony. The tweets that created more impact were published by news sources or by sports reporters. Lastly, the themes that emerged showed that the Los Angeles Lakers and the NBA were the two most important milestones in Pau Gasol’s career. The data can be used to detect potential areas of controversy or other issues to be addressed in order to preserve the athlete’s public image. These results are considered of interest for reaching better knowledge of sport virtual environments through social sensing, supporting the idea of users acting as sensors.

## 1. Introduction

Social media is a significant mode of communication all over the world and plays an important role in all aspects of life [1]. It has entered social lives, affecting not only social relations on the internet but also in the physical world in areas such as the labor sector [2], economic sector [3,4], or health [5], among others [6].

Since 2006, Twitter has grown exponentially. In 2019, the platform was reported to have around 330 million active users [7] generating 500 million tweets per day [8]. As of the first quarter of 2019, Twitter no longer reports active users. Even so, some sources inform that as of December 2022, Twitter accounts for over 368 million active users, although this figure is projected to decrease by 5% in the following two years [9], coinciding in terms of time with Elon Musk’s purchase of the platform. It is a microblogging platform that allows users to share opinions, news, or other facts within a limit of 280 characters. As there is a huge amount of information provided by Twitter, very diverse analytic approaches have emerged, as a previous systematic review identified, including content, sentiment analysis, image analysis, surveillance, prediction, engagement, or network analysis [5].

Among the types of Twitter analysis, we also find quantitative and qualitative methods [10]. Quantitative analysis allows understanding the traffic of tweets during a particular time. User reactions to specific events can be monitored and interpreted. In this regard, word frequency, number of mentions, and number of retweets are of high interest. Qualitative analysis is usually performed through content analysis [11], which makes it possible to study the meaning of the messages. The combination of quantitative and qualitative methods has been identified by some authors as a useful complementary research technique, since it allows a combination of technical capabilities of analysis with in-depth qualitative research methods [12]. Quantitative analysis makes information available at the macrolevel, whereas qualitative analysis helps to interpret the microlevel [13]. Support of this combination of methods is made by previous researchers and considered necessary for the knowledge of large social databases [11,13].

One of the approaches to using Twitter in research is understanding social sensing [14]. In social sensing, individuals play the role of a sensor, which makes it possible to understand collective environments. Social sensing can even include richer information than traditional remote sensing data [15]. For example, social media allow the understanding of spatial interactions and semantic uses through messages or shared content.

Extracting data from platforms where individuals share content, such as social media, allows insights into real-world systems [14]. The data that social sensing can benefit from is diverse, and includes, for instance, commuting trajectories, trips on public transportation systems, mobile phone records, smart card records, social media, and social networking data, among others [16].

Social sensing has traditionally been applied in different fields of knowledge. Politics is the most common area of interest from the social sensing perspective [17,18]. However, analysis and even prediction of crime [19], natural disasters [20], or other hazards [21] as well as health [22] and meteorology [14] have also shown interest in this topic. Fake news and misinformation have also been topics of interest in previous social sensing research [23,24].

Sports, as a worldwide phenomenon, have also shown advances in social sensing during the last decade [25,26,27]. Specifically, Twitter has become an integral part of sport media [28]. It has become a tool for fan engagement and for enhancing individual-level brands and other related brands (e.g., organization-level brands such as sport leagues’ brands). Twitter and other social media platforms have changed the sports landscape, creating new social networks and parallel backchannel conversations. In this regard, interactions between professional sports teams and fans [29], athletes’ use of social media for self-promotion [30], international federations [31], and sponsor activations [32] has been studied. Specific sporting events have also been researched [29], as well as the public response to specific situations during sporting events, such as the Boston marathon explosions in 2013 [33]. The advance in the knowledge of sports brands would help to identify opportunities to understand how individual-level brands (e.g., athletes) and organization-level brands (e.g., sport leagues) may use social media and interact with fans [34].

One of the most socially commented leagues is the National Basketball Association (NBA). The NBA is the most internationally recognized brand and the most successful sports league in the world [35]. During the 2021/2022 season, the NBA experienced 135 million NBA-related tweets from 6.5 million unique users (+ 24% tweets and + 34% users compared to the previous season) [36]. One of the most influential players of the last two decades is Pau Gasol, having had his jersey number (16) retired by the Los Angeles Lakers as a sign of accomplishment [37]. He started playing basketball in Spain (his birthplace) and he joined the NBA in 2001. Since then, he has won two NBA championships. He has played on five NBA teams: the Memphis Grizzlies, the Los Angeles Lakers, the Chicago Bulls, the San Antonio Spurs, and the Milwaukee Bucks. He has also won a world championship (2006), three European championships (2009, 2011, and 2015), and three Olympic medals (silver in Beijing 2008, silver in London 2012, and bronze in Rio de Janeiro 2016). All these career accomplishments have led to the development of an individual-level brand. In February 2021, Pau Gasol returned to FC Barcelona, which was his first professional basketball team. Later on October 5, the 41-year-old player announced his retirement from professional basketball, ending a successful and exemplary career [38].

Although many scientific articles have been published on sports social media analysis [1,29,39,40,41], not enough research has been completed on the effects of sport individual-level brands after their active disassociation from the competition. Pau Gasol’s retirement announcement can be considered an event that was commented on and observed internationally. The main research contribution of this study is the exploration of an individual-level brand that is a reference internationally. Understanding and identifying how social sensing behaves can be useful for understanding how individual-level brands, such as Pau Gasol’s personal brand, influences and is influenced by other related brands, such as the NBA or the different NBA teams, as part of the legacy.

Thus, this paper aims to explore the content of English-language reactions on Twitter to quantitatively and qualitatively analyze the social sensing linked to an individual-level brand. For this purpose, the case of Pau Gasol’s retirement announcement will be analyzed.

## 2. Materials and Methods

### 2.1. Sampling Strategy

This research explores tweets published before, during, and after Pau Gasol’s public retirement announcement. Twitter posts were explored from Tuesday 5 October 2021 at 3:00 to Thursday 7 October 2021 at 23:59, Greenwich Mean Time (GMT), +00:00 time zone. The selected study period is considered to be wide enough to monitor the opinions of internet users about the topic of study. It comprises 12 h before Pau Gasol’s retirement announcement, which was held on Tuesday 5 October 2021 at 15:00 (GMT +00:00), and the two following days after the announcement.

All the tweets during this period were collected and stored 8 days after the farewell (13 October 2021 at 16:56, GMT +00:00). This window of time is small enough for ensuring all the tweets published during those days were retrieved and no message was lost, and it was large enough to allow users to spontaneously interact with the posted messages (e.g., retweeting).

### 2.2. Data Extraction and Collection

Data collection was performed through QSR NVivo software. A single search was completed on Wednesday 13 October 2021 at 16:56 (GMT +00:00). However, a double-check with previous preliminary searches was performed for avoiding data loss. Additionally, random checks were completed for ensuring the tweets collected were reliable. The search strategy was as follows:

- Tweets should include at least one of the following words or hashtags: “Gasol” OR “#GraciasPau” OR #ThankYouPau” OR “#PauGasol”. Since #GraciasPau and #ThankYouPau were the most common hashtags on the topic and the Trending Topic at that moment, it is reasonable to consider that almost all the tweets contained at least one of these words or hashtags. The hashtag #PauGasol, including the full name of the player, was not a Trending Topic at that moment, but nevertheless, it was a common hashtag in the posts. It was included in the search query, since its non-inclusion would have meant a significant loss of relevant data related to the event. The word ‘Gasol’ was also part of the search query because it is the name by which he is most commonly known in the scope of basketball. All the previous allowed the retrieval of tweets about Pau Gasol without the risk of collecting posts linked to another different topics. For example, a pilot test with the term “Pau” was performed, but multiple tweets about diverse topics emerged (for instance, tweets related to acronyms of P.A.U.).
- Only original tweets were included. No retweets were added to the database. Thus, no duplicated posts are contained in the record.
- Only tweets written in the English language were considered, due to it being considered the lingua franca on Twitter [11,42].
- Replies of original tweets were also not included in the study.
- Duplicated messages were merged.

Therefore, the search query was:

(Gasol OR #GraciasPau OR #ThankYouPau OR #PauGasol) lang:en -filter:replies -filter:retweets until:2021-10-08 since:2021-10-05.

After retrieving all the tweets, a deep preprocessing work was made, following recommendations in previous research [43]. URL links in the corpus were removed for reducing the amount of noise in the tweets, as suggested in previous literature [11,43]. For example, the text “https” and “https://” was completely removed before the data analysis. All the words empty of meaning were removed. For example, stop words such as “the”, “and”, and “at” were cleaned out.

### 2.3. Data Analysis

A combination of quantitative and qualitative methods was conducted in this research. Content analysis was performed for analyzing the received tweets.

QSR NVivo software was used for quantitative analysis, as suggested in previous sport-related research [44]. This software collects tweets through an add-on called NCapture. Then, all tweets are transferred to QSR NVivo software for analysis. Following previous literature [31,45], the quantitative results were presented as frequencies of tweets, retweets, users, mentions, followers, and word frequency. Betweenness centrality was also analyzed to identify the most influential accounts posting about the event. IBM SPSS 29.0 Statistics software (IBM Inc., Chicago, IL, USA) was used for complementary quantitative data analysis, such as the histogram of retweets.

Qualitative analysis was also performed through QSR NVivo. Theme analysis and message codification were performed. For qualitative word analysis, only words with three or more letters were considered. Derived words were grouped together (for instance, “talk” and “talking”). QSR NVivo allows grouping words following one of these five levels: exact matches (e.g., “talk”), with stemmed words (e.g., “talking”), with synonyms (e.g., “speak”), with specializations (e.g., “whisper”), or with generalizations (e.g., “communicate”). In this specific case, grouping with stemmed words was chosen to avoid the loss of hints due to non-controlled grouping and because it was considered to be more interpretable.

## 3. Results

### 3.1. Quantitative Analysis

A total of 2089 tweets were recovered from Tuesday 5 October 2021 at 3:00 to Thursday 7 October 2021 at 23:59. All hours are expressed following Greenwich Mean Time (GMT), +00:00 time zone. The tweets retrieved were original tweets, meaning that they were not retweets of previous tweets.

**Figure 1:**
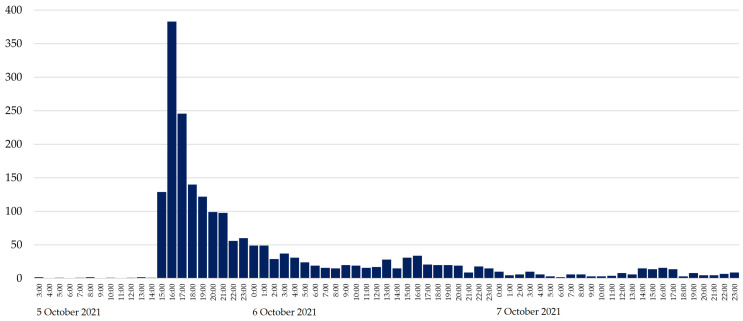
Tweet distribution by hour. Period of study: from Tuesday 5 October 2021 at 3:00 to Thursday 7 October 2021 at 23:59 (Greenwich Mean Time, GMT, +00:00 time zone).

Figure 1 shows the tweet traffic distribution during this period regarding messages related to the retirement announcement by Pau Gasol, following a left-skewed distribution. The farewell started at 15:00 on 5 October 2021, coinciding with the first big growth in number of tweets (*n* = 129). However, the peak in the number of tweets was obtained on 5 October 2021 between 16:00 and 17:00 (*n* = 383).

**Figure 2:**
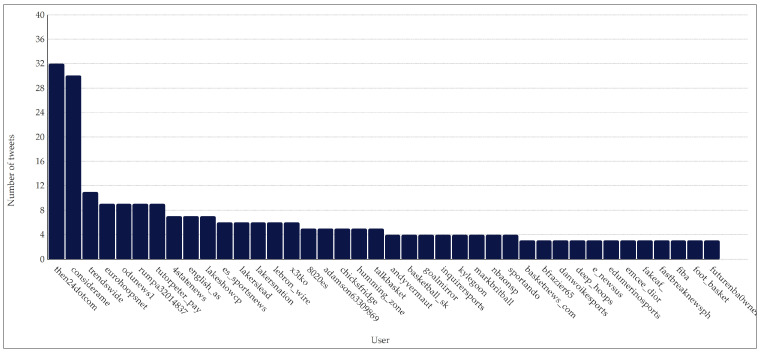
Number of tweets per user on Pau Gasol’s retirement announcement.

There were two main users that together covered 2.96% of the total tweets retrieved. @then24dotcom published 32 tweets (coverage of 1.53%) and @considerame published 30 tweets (coverage of 1.43%). The user who tweeted the third-largest number of tweets was @trendswide, with 11 tweets (coverage of 0.53%). The rest of the users published nine tweets or less, meaning that their individual coverage was 0.43% or less. Figure 2 shows a power-law distribution of the 40 users with the most published tweets about Pau Gasol’s farewell.

**Figure 3:**
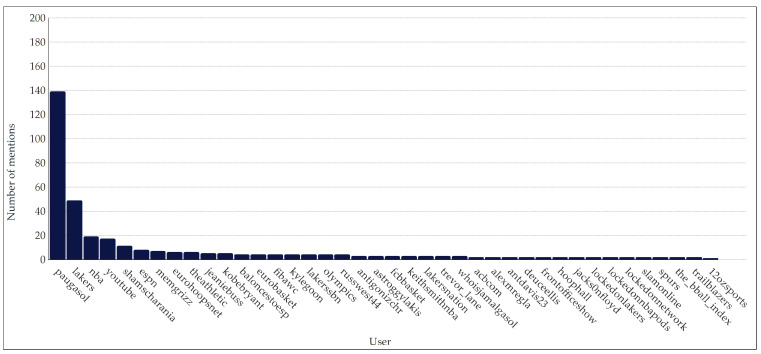
Number of mentions per user on Pau Gasol’s retirement announcement.

Regarding the most-mentioned users (Figure 3), @paugasol leads the ranking with 139 direct mentions (coverage of 6.63%), followed by @lakers with 49 tweets (2.34% coverage), and @NBA with 19 tweets (0.80% coverage). The following accounts are @youtube (*n* = 17), @ShamsCharania (*n* = 11), @espn (*n* = 8), @memgrizz (*n* = 7), @eurohoopsnet (*n* = 6), @theathletic (*n* = 6), @jeaniebussn (*n* = 5), and @kobebryant (*n* = 5). A power-law distribution can be observed.

**Figure 4:**
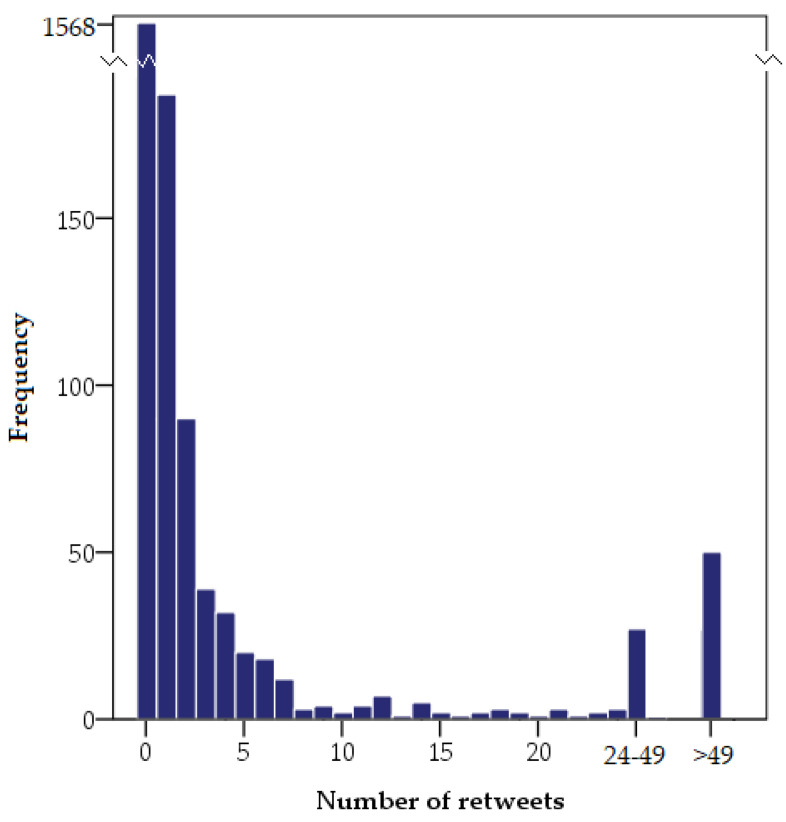
Number of retweets of the collected tweets.

The most shared tweet was published by @ShamsCharania and was retweeted 6705 times, followed by the one published by @BleacherReport (3843 retweets) and the tweet by @espn (3779 retweets). The verbatim transcriptions of the three most shared tweets are shown in Table 1. Table 2 shows the users with messages retweeted by more than 1000 users. The only user that appears twice in the list is @ShamsCharania, senior lead NBA insider, writer, and analyst. Regarding the date and hour, the five most shared tweets were published on 5 October 2021 between 15:43 and 17:14, during and right after the farewell. Figure 4 shows the frequency of retweets during the study period. A total of 1568 tweets (75.06%) were not shared, whereas 521 tweets (24.94%) were shared at least once. During that time, 27 messages (1.29%) were retweeted between 25 and 49 times, and 50 messages (2.39%) were retweeted more than 50 times.

The most shared tweets (over 1000 retweets) came mainly from two sources: new sources specializing in sports (@BleacherReport, @espn, @SportsCenter) or basketball (@Eurohoopsnet, @overtime, @ESPNNBA). Also among them were noticeable sports reporters (@ShamsCharania), television programs focused on basketball (@NBAonTNT), associations (@NBA), and Twitter accounts offering viral information (@LakeShowYo).

Table 3 shows the betweenness centrality results of the sample. This is interpreted as the Twitter users that acted as ‘bridges’ to other Twitter users in the conversation on Pau Gasol’s retirement announcement. As observed, Pau Gasol was the main bridge in the conversation, followed by the NBA team Los Angeles Lakers. This point connects with the qualitative analysis.

### 3.2. Qualitative Analysis

The qualitative analysis was performed considering the different themes that emerged in the tweets (Table S1: Word topics). Thus, Gasol (or #gasol) and Pau (or #pau) were common terms since those words were part of the search query. These terms were mainly mentioned by news sources, but also as hashtags. They were also highly common together (PauGasol) or as a mention to the player himself (@PauGasol).

The acronym NBA for National Basketball Association (or #nba, @nba) was also one of the most common terms in the collected tweets. This word was very frequently together with other terms, creating bigrams such as ‘NBA champion’, ‘NBA championship’, and ‘NBA titles’. Some users used the trigram ‘two NBA championships’, referring to the two titles earned by Pau Gasol with the Los Angeles Lakers. Less common was the recall to the NBA rookie of the year earned by Gasol in 2002, being the first Spaniard with that distinction. Although Pau Gasol played his last months in his first professional basketball club, FC Barcelona, these results evince that the NBA was the competition that introduced Pau Gasol internationally.

Additional frequent terms referred to retirement (also #retirada, #retire, #retired, #retires, retira, retirada, retiradas, retire, retired, retires, retiro, or #retirement). This information appeared mainly in informative tweets but not as commonly in emotional messages posted by fans. This word was frequently together with ‘Pau Gasol’ and was used very commonly as a hashtag.

The phrase Los Angeles Lakers (or #lakers, @lakers, lakers) was less common but still prevalent among users. Noticeably, although Pau Gasol left the Los Angeles Lakers in 2014, it was the most commonly mentioned team on Twitter. A considerable number of messages highlighted that Los Angeles Lakers are planning to retire number 16 from their team, which matches with the bib number of Pau while playing on that team. Some tweets also recalled the role that Kobe Bryant played on the team together with Pau Gasol.

Basketballer (or #basketball, basketball, basketballer) was commonly used together with words derived from retirement.

The word career was also recurrent. Users posting on this topic used this term mainly in two ways: referring to his ‘ending career’ or together as a positive adjective, showing positive sentiment (for instance, ‘amazing career’).

The topic referring to the announcement (words such as announces, announce, or announced) was commonly used by news sources for headlining the news (for example, ‘Pau Gasol officially announces his retirement’). The word time (or times) was usually applied for listing his honors, with the number of won championships (for instance, ‘Two-time NBA champion).

Finally, the memory of Kobe Bryant remains in the minds of Pau Gasol’s and the NBA’s fans. The word Kobe was frequently mentioned by users, something that was motivated, in part, by the tribute that Pau dedicated to Kobe during the retirement announcement.

**Figure 5:**
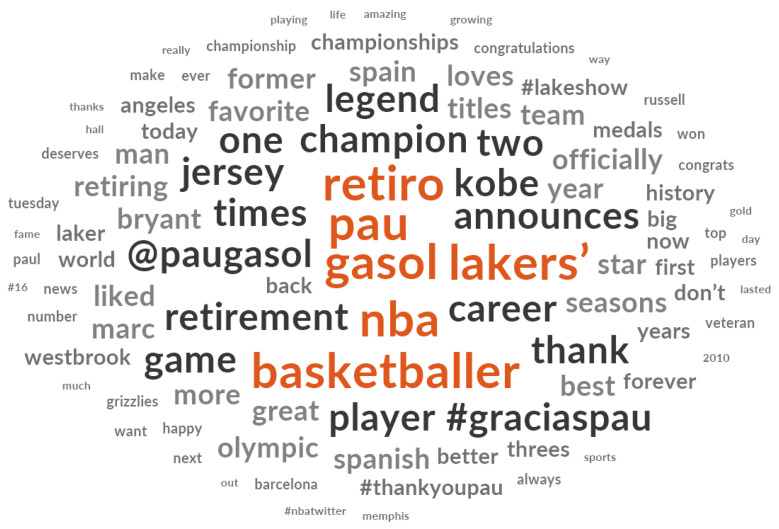
Word cloud of the topics emerged.

For easier interpretation of the topics that emerged, a word cloud is shown in Figure 5. It represents frequently occurring words in the analyzed data. The bigger and bolder a word is, the more frequent it was in the collected messages. Moreover, the orange color helps to differentiate the more frequent words, whereas the grey color represents the less frequent words. Thus, the obtained word cloud facilitates the interpretation of the previous results.

## 4. Discussion

This research contributes to the application of Twitter messages as social sensors. The case of Pau Gasol’s retirement announcement shows how his individual brand influences and is influenced by other related brands. Pau Gasol’s retirement announcement is an internationally commented event that can help to understand social sensing in sports situations, but it also has implications in other contexts. For example, brands from other industries could apply these methods to analyze reactions to their marketing campaigns. Furthermore, public figures from outside of the sports world may find it useful to explore the reactions on Twitter to increase their impact or reinforce specific messages.

First, the phenomenon studied has been explored from the perspective of international fans. This means that the results should be interpreted from the context of an English speaking Twitter user. Some authors suggest that English is considered the lingua franca on Twitter [11,42]. Although no recent research exists about the language use in social media, explorations dating from 2011 and 2012 identified English as the most popular language, representing between 51% [46] and 73% of the total messages [42].

The quantitative analysis measured the impact of conversations on Pau Gasol’s retirement announcement on Twitter. The increase in tweet traffic was stimulated by the starting hour of Pau Gasol’s farewell. The event started at 15:00 on 5 October 2021, but the peak was obtained at 16:00 that same day (GMT +00:00). This means a growth of a 197% in one hour. In the following hours, the number of tweets progressively decreased. The previously mentioned decrease of 197% took three hours. This fast-growing traffic contrasts with a relatively slower decrease compared to the growth rate, something that has also been observed in previous research on Twitter [11].

The messages with more retweets (more than 1000 retweets) were posted by news sources, showing coherence with previous research on political journalists [47]. Therefore, Twitter users face information that is merely informative. The potential of Twitter as a news media has been discussed previously in some work [48,49], but this new evidence may reinforce that social sensing might be highly influenced by news sources’ users and pundits, as it happens with traditional media (television, radio, or newspapers). These users channel and direct the attention of fans and followers to their posts and shared content. Although the analyzed event in this research does not deal with highly sensitive information, the potential risks of misinformation from certain Twitter users could be higher in the case of topics such as health [23] or politics [24]. Specific users acting as influential (or hub users) has been previously observed in sporting events [26]. For that reason, the quantitative aspect (number of tweets) should be interpreted cautiously. This idea reinforces the usefulness of combining quantitative and qualitative methods in Twitter social sensing analysis.

Regarding the most mentioned users after @PauGasol, @Lakers and @NBA take the first and second place. Despite Pau Gasol stopping his contract with the Los Angeles Lakers in 2014, it was the team where he reached the greatest success, and the NBA was the league that projected him internationally. Although the number of mentions is not apparently connected to the number of retweets, some authors suggest that there is a relationship between them, for example, with vulnerability, user online status, and location similarity [50]. Thus, the probability of a user retweeting a message, posting updated information, and tweets surrounding users positively influences the probability of being retweeted. In this study, the most shared messages were posted on 5 October 2021 between 15:43 and 17:14, during and after the farewell, which meets the criteria of instant information, avoiding outdated messages. However, this study’s results differ from the other two criteria. The most posted tweets did not usually include mentions, contrasting with the analysis by Li et al. [50]. Additionally, as Pau Gasol’s farewell had an international impact, these retweets had not only had a regional impact, but global.

By the betweenness data of users’ accounts and the qualitative analysis, it can be observed that the main bridges between Twitter users were an individual-level brand, @paugasol, and a group brand, @Lakers. After them, the two following were reporters or pundits (@RoseSportsPod; @ShamsCharania). By complementing the quantitative analysis with the qualitative analysis, it was observed that the main users (the previously mentioned users) did not provide clear opportunities for the audience to interact with their messages apart from with retweets. Clearly dominated networks also exist in other contexts, such as in politics [47]. There, political journalists were observed to act as the expert talking rather than encouraging users to participate in a discussion. This reality has some risks, such as the danger of biased information and misinformation, having also been reported in previous work [26].

The qualitative analysis shows that the themes that emerged in the conversations were related to the protagonist, Pau Gasol, and the reason for the event, his retirement. One remarkable moment in the Twitter conversation was remembering Kobe Bryant’s memory. The reason for this may be linked to emotional aspects. The memory of Kobe Bryant can be considered one of the most emotional moments of the analyzed event. In previous research, some authors identified that Twitter users usually immediately react to both positive and negative events through bursts of tweets, but positive events are more likely to induce a growth of traffic [26]. Specially interesting is the discussion of whether the mention to Kobe Bryant’s memory was a positive or a negative episode. The results obtained could make us think that Pau Gasol’s mentioning of Kobe Bryant in a loving and affectionate way was a positive episode, while the accident suffered by the latter in 2020 was not.

Another remarkable circumstance was that the NBA and the Los Angeles Lakers were in the first positions of topics discussed, although, as previously discussed, Pau Gasol’s role in the Los Angeles Lakers finished in 2014, and he does not belong to the NBA anymore. This fact is closely related to the called unaided recall or spontaneous recall, very common in branding [51,52]. When users talk about certain topics or when they are asked about them, their mind automatically recalls other words, people, or situations. Interestingly, some of some words, people, or situations are not necessarily accurate. This is the case of this research, where Pau Gasol’s individual-level brand was unconsciously linked to brands such as the Los Angeles Lakers or the NBA, which are not associated anymore. Unaided recall has been explored in sports, mainly in brands and sponsorship studies [53]. In previous sports-related works, the convenience of using sports stars’ personal brands on global social media as a means to craft and maintain global brand equity and increasing brand awareness has been proven [54]. Thus, a long-lasting sportsman’s or sportswoman’s career contributes to creating their own brand all through their professional trajectory. In this study, this fact is well-observed through spontaneously posted messages and through the betweenness of Twitter users.

## 5. Conclusions

This analysis explores, through Pau Gasol’s retirement case, how people posting messages and acting as social sensors provides knowledge about an individual-level brand and its relation to other brands. Social sensing was analyzed through individual messages posted on Twitter right before, during, and right after the ceremony. Thus, Twitter can be understood as a network of individual sensors.

The results indicate the following conclusions. First, the tweet publication was almost limited to the duration of the farewell. However, the volume of tweets should be interpreted cautiously in Twitter social sensing analysis since there are a few users (also called hub users) that accumulated most of the tweets. Second, news sources users are profiles that create a big impact on Twitter. These users act as influencers for individuals, who share their content and cause data bias. Similar are the profiles of sport reporters, television programs, or associations. Third, the brand interaction was not necessarily accurate since Pau Gasol’s individual-level brand affects and is affected not only by current related brands but by previous milestones. For instance, the Los Angeles Lakers emerged in this research as a common topic, but in some way, it is not accurate to the current time since the interaction between both brands is not current. These results are considered potentially interesting for better understanding virtual environments in the sports field through social sensing, thanks to individual users acting as sensors.

One of the main practical contributions of the research is the management of public exhibition of an international athlete, such as a press conference. By monitoring the reactions on social media, the athlete and their staff can gain insight into how the public perceives their comments and behavior, allowing them to adjust their public statements and conduct accordingly in order to maximize impact, minimize the risk of misinterpretation, and efficiently connect with a large fan base. Additionally, managers can use the data to tailor their strategy for future press conferences, if necessary. Finally, the data can also be used to identify potential areas of controversy or other potential issues that need to be addressed in order to maintain the athlete’s public image.

This study deals with some limitations. First, only messages in English language were collected, as it is the lingua franca on Twitter. This was made following recommendations of previous research [11,42]. This allowed an international analysis, with international brands related to the NBA, instead of a local analysis, which is more geographically limited. Thus, knowing that the conclusions are limited by the language of the tweets, an analysis with tweets published in Spanish might have led to different conclusions, but more limited geographically and culturally. Second, this study explores social sensing only via Twitter. The messages posted could differ from other social media or even from face-to-face communication.

Future lines of research could focus on analyzing social sensing in similar sporting events or ceremonies according to different languages. For example, events such as the one explored in this article could offer diverse insights when analyzing messages in languages such as English, Spanish, or Chinese.

## Figures and Tables

**Table 1:** Verbatim transcriptions of the three most shared tweets.

User	Verbatim Transcription of the Tweet
@ShamsCharania	The Los Angeles Lakers plan to retire Pau Gasol’s No. 16 jersey
@BleacherReport	Pau Gasol has retired after 19 NBA seasons and two championships [Picture of Pau Gasol in the retirement press conference]
@Espn	In his retirement press conference, Pau Gasol reflected on his relationship with Kobe Bryant [Clapping hands sign emoji] [Split screen image with a picture of Pau Gasol in the retirement press conference, picture from behind of Pau Gasol holding Kobe Bryant with affection, and the quote ‘I want to make a special mention to Kobe Bryant. I’d very much like him to be here but that’s life. He taught me how to be a better leader, better competitor, what if meant to be a winner. Pau Gasol on Kobe Bryant while announcing his retirement’]

**Table 2:** Most retweeted messages on Pau Gasol’s retirement announcement.

User	Number of Followers	Number of Retweets	Date and Hour ^1^
@ShamsCharania	1,425,041	6705	10/05/2021 16:16:47
@BleacherReport	10,146,731	3843	10/05/2021 15:48:56
@Espn	67,660,299	3779	10/05/2021 17:14:25
@ShamsCharania	1,425,041	3520	10/05/2021 15:43:14
@Eurohoopsnet	84,517	2999	10/05/2021 16:06:44
@SportsCenter	38,221,637	2392	10/05/2021 16:34:01
@Overtime	954,689	2245	10/05/2021 15:46:57
@NBAonTNT	4,588,719	1843	10/05/2021 15:51:04
@NBA	34,657,056	1470	10/05/2021 19:34:00
@LakeShowYo	184,923	1237	10/05/2021 19:12:19
@ESPNNBA	7,189,364	1122	10/05/2021 16:44:14

^1^ Date and hour format: month/day/year hour:minute:second (MM/DD/YYYY HH:MM:SS). (Greenwich Mean Time, GMT, +00:00 time zone).

**Table 3:** Betweenness of user accounts.

User	Betweenness
@paugasol	58.98
@Lakers	26.63
@RoseSportsPod	9.25
@ShamsCharania	8.99
@Youtube	8.53
@jugonness	6.93
@ZSPN_Sports	6.50
@LakersHeritage	6.17
@Hoophall	4.79
@deephoops	4.49
@LakersSBN	4.34
@THEMcGodiva	4.27
@espn	3.79

## Data Availability

All data are publicly available in Twitter. Specific data are also available in Supplementary Materials.

## Supplementary Materials

The following supporting information can be downloaded at: https://www.mdpi.com/article/10.3390/ijerph20020895/s1, Table S1: Word topics.

## References

[1] Wang Y., Zhou S. (2015). How Do Sports Organizations Use Social Media to Build Relationships? A Content Analysis of NBA Clubs’ Twitter Use. Int. J. Sport Commun..

[2] van Zoonen W., Verhoeven J.W.M., Vliegenthart R. (2016). How employees use Twitter to talk about work: A typology of work-related tweets. Comput. Hum. Behav..

[3] Prada A., Iglesias C.A. (2020). Predicting Reputation in the Sharing Economy with Twitter Social Data. Appl. Sci..

[4] Wei W., Mao Y., Wang B. (2016). Twitter volume spikes and stock options pricing. Comput. Commun..

[5] Sinnenberg L., Buttenheim A.M., Padrez K., Mancheno C., Ungar L., Merchant R.M. (2017). Twitter as a Tool for Health Research: A Systematic Review. Am. J. Public Health.

[6] Noor S., Guo Y., Shah S.H.H., Nawaz M.S., Butt A.S. (2020). Research Synthesis and Thematic Analysis of Twitter through Bibliometric Analysis. Int. J. Semant. Web Inf. Syst..

[7] Statista Number of Monthly Active Twitter Users Worldwide from 1st Quarter 2010 to 1st Quarter 2019. https://www.statista.com/statistics/282087/number-of-monthly-active-twitter-users/.

[8] Sayce D. The Number of Tweets per Day in 2020. https://www.dsayce.com/social-media/tweets-day/.

[9] Statista Twitter: Number of Worldwide Users 2019–2024. https://www.statista.com/statistics/303681/twitter-users-worldwide/.

[10] Gelfgren S., Griffin G., Hayler M. (2016). Reading Twitter: Combining Qualitative and Quantitative Methods in the Interpretation of Twitter Material. Research Methods for Reading Digital Data in the Digital Humanities.

[11] Millán-González L., Devís-Devís J., Pellicer-Chenoll M., Pans M., Pardo-Ibañez A., García-Massó X., Peset F., Garzón-Farinós F., Pérez-Samaniego V. (2021). The impact of COVID-19 on sport in twitter: A quantitative and qualitative content analysis. Int. J. Environ. Res. Public Health.

[12] Tinati R., Halford S., Carr L., Pope C. (2014). Big Data: Methodological Challenges and Approaches for Sociological Analysis. Sociology.

[13] Blok A., Pedersen M.A. (2014). Complementary social science? Quali-quantitative experiments in a Big Data world. Big Data Soc..

[14] Young J.C., Arthur R., Spruce M., Williams H.T.P. (2021). Social Sensing of Heatwaves. Sensors.

[15] Liu Y., Liu X., Gao S., Gong L., Kang C., Zhi Y., Chi G., Shi L. (2015). Social Sensing: A New Approach to Understanding Our Socioeconomic Environments. Ann. Assoc. Am. Geogr..

[16] Lu Y., Liu Y. (2012). Pervasive location acquisition technologies: Opportunities and challenges for geospatial studies. Comput. Environ. Urban Syst..

[17] Budiharto W., Meiliana M. (2018). Prediction and analysis of Indonesia Presidential election from Twitter using sentiment analysis. J. Big Data.

[18] Yaqub U., Chun S.A., Atluri V., Vaidya J. (2017). Analysis of political discourse on twitter in the context of the 2016 US presidential elections. Gov. Inf. Q..

[19] Williams M.L., Burnap P., Sloan L. (2016). Crime Sensing with Big Data: The Affordances and Limitations of using Open Source Communications to Estimate Crime Patterns. Br. J. Criminol..

[20] Pourebrahim N., Sultana S., Edwards J., Gochanour A., Mohanty S. (2019). Understanding communication dynamics on Twitter during natural disasters: A case study of Hurricane Sandy. Int. J. Disaster Risk Reduct..

[21] Poblete B., Guzman J., Maldonado J., Tobar F. (2018). Robust Detection of Extreme Events Using Twitter: Worldwide Earthquake Monitoring. IEEE Trans. Multimed..

[22] Edo-Osagie O., De La Iglesia B., Lake I., Edeghere O. (2020). A scoping review of the use of Twitter for public health research. Comput. Biol. Med..

[23] Kouzy R., Abi Jaoude J., Kraitem A., El Alam M.B., Karam B., Adib E., Zarka J., Traboulsi C., Akl E., Baddour K. (2020). Coronavirus Goes Viral: Quantifying the COVID-19 Misinformation Epidemic on Twitter. Cureus.

[24] Bovet A., Makse H.A. (2019). Influence of fake news in Twitter during the 2016 US presidential election. Nat. Commun..

[25] Takeichi Y., Sasahara K., Suzuki R., Arita T. (2014). Twitter as Social Sensor: Dynamics and Structure in Major Sporting Events. Proceedings of the Artificial Life 14: Fourteenth International Conference on the Synthesis and Simulation of Living Systems.

[26] Takeichi Y., Sasahara K., Suzuki R., Arita T. (2015). Concurrent Bursty Behavior of Social Sensors in Sporting Events. PLoS ONE.

[27] Vasudevan V., Wickramasuriya J., Zhao S., Zhong L. (2013). Is Twitter a good enough social sensor for sports TV?. Proceedings of the 2013 IEEE International Conference on Pervasive Computing and Communications Workshops (PERCOM Workshops).

[28] Gibbs C., Haynes R. (2013). A Phenomenological Investigation Into How Twitter Has Changed the Nature of Sport Media Relations. Int. J. Sport Commun..

[29] Yan G., Watanabe N.M., Shapiro S.L., Naraine M.L., Hull K. (2019). Unfolding the Twitter scene of the 2017 UEFA Champions League Final: Social media networks and power dynamics. Eur. Sport Manag. Q..

[30] Taniyev O., Ishaq F., Gordon B. (2018). Investigating the Differences in Twitter Content and Effectiveness Between Individual and Team Sport Athletes. Int. J. Bus. Adm..

[31] Winand M., Belot M., Merten S., Kolyperas D. (2019). International Sport Federations’ Social Media Communication: A Content Analysis of FIFA’s Twitter Account. Int. J. Sport Commun..

[32] Eddy T., Cork B.C., Lebel K., Howie Hickey E. (2021). Examining Engagement With Sport Sponsor Activations on Twitter. Int. J. Sport Commun..

[33] Cassa C.A., Chunara R., Mandl K., Brownstein J.S. (2013). Twitter as a Sentinel in Emergency Situations: Lessons from the Boston Marathon Explosions. PLoS Curr..

[34] Geurin A.N., Burch L.M. (2017). User-generated branding via social media: An examination of six running brands. Sport Manag. Rev..

[35] Schwarz E.C., Dodds M., Heisey K., Ahonen A. (2018). Marketing implications of playing regular season games in international markets. Routledge Handbook of International Sport Business.

[36] Twitter Marketing The Conversation: NBA & WNBA Are a Slam Dunk on Twitter. https://marketing.twitter.com/en/insights/twitter-conversation-report-basketball-2022.

[37] NBA Pau Gasol Retires, Lakers Reportedly Planning to Hang up No. 16. https://www.nba.com/news/reports-lakers-plan-to-retire-pau-gasols-jersey.

[38] ESPN Pau Gasol, Two-Time NBA Champion, Announces Retirement from Basketball. https://www.espn.com/nba/story/_/id/32343088/veteran-pau-gasol-announces-retirement-basketball.

[39] Abeza G., O’Reilly N., Seguin B. (2019). Social Media in Relationship Marketing: The Perspective of Professional Sport Managers in the MLB, NBA, NFL, and NHL. Commun. Sport.

[40] Abeza G., O’Reilly N., Seguin B., Nzindukiyimana O. (2017). Social Media as a Relationship Marketing Tool in Professional Sport: A Netnographical Exploration. Int. J. Sport Commun..

[41] Abeza G., O’Reilly N., Reid I. (2013). Relationship Marketing and Social Media in Sport. Int. J. Sport Commun..

[42] Takhteyev Y., Gruzd A., Wellman B. (2012). Geography of Twitter networks. Soc. Netw..

[43] Jianqiang Z., Xiaolin G. (2017). Comparison Research on Text Pre-processing Methods on Twitter Sentiment Analysis. IEEE Access.

[44] Corbett B., Edwards A. (2018). A case study of Twitter as a research tool. Sport Soc..

[45] Watkins B. (2018). Sport Teams, Fans, and Twitter. The Influence of Social Media on Relationships and Branding.

[46] Hong L., Convertino G., Chi E. (2011). Language Matters In Twitter: A Large Scale Study. Proc. Int. AAAI Conf. Web Soc. Media.

[47] Nuernbergk C. (2016). Political Journalists’ Interaction Networks. J. Pract..

[48] Broersma M., Graham T. (2013). Twitter as a news source: How Dutch and British newspapers used tweets in their news coverage, 2007–2011. J. Pract..

[49] Seth A., Nayak S., Mothe J., Jadhay S. (2017). News Dissemination on Twitter and Conventional News Channels. Proceedings of the 19th International Conference on Enterprise Information Systems.

[50] Li Y., Ding Z., Zhang X., Liu B., Zhang W. (2016). Confirmatory Analysis on Influencing Factors When Mention Users in Twitter. Web Technologies and Applications: Proceedings of the Web Technologies and Applications APWeb 2016 Workshops, WDMA, GAP, and SDMA, Suzhou, China, 23–25 September 2016.

[51] Maricic M., Kostic-Stankovic M., Bulajic M., Jeremic V. (2019). See it and believe it? Conceptual model for exploring the recall and recognition of embedded advertisements of sponsors. Int. J. Sport. Mark. Spons..

[52] Tan S.Y., Pyun D.Y. (2018). The Effectiveness of Sponsorship of the F1 Singapore Grand Prix. Int. J. Asian Bus. Inf. Manag..

[53] Quester P.G. (1997). Awareness as a measure of sponsorship effectiveness: The Adelaide Formula One Grand Prix and evidence of incidental ambush effects. J. Mark. Commun..

[54] Zhou F., Mou J., Su Q., Jim Wu Y.C. (2020). How does consumers’ Perception of Sports Stars’ Personal Brand Promote Consumers’ brand love? A mediation model of global brand equity. J. Retail. Consum. Serv..

